# Identifying Complex Scheduling Patterns Among Patients With Cancer With Transportation and Housing Needs: Feasibility Pilot Study

**DOI:** 10.2196/57715

**Published:** 2025-01-17

**Authors:** Allan Fong, Christian Boxley, Laura Schubel, Christopher Gallagher, Katarina AuBuchon, Hannah Arem

**Affiliations:** 1 MedStar Health Research Institute, 3007 Tilden St, Washington, DC, 20008, United States, 1 202-244-9807; 2 MedStar Washington Hospital Center, Washington, DC, United States; 3 Lombardi Comprehensive Cancer Center, MedStar Georgetown University, Washington, DC, United States

**Keywords:** patient scheduling, scheduling complexities, temporal data mining, dataset, breast cancer, social determinant of health, oncology, metastasis, cancer patient, social support, community health worker, housing need, care, transportation, algorithm

## Abstract

**Background:**

Patients with cancer frequently encounter complex treatment pathways, often characterized by challenges with coordinating and scheduling appointments at various specialty services and locations. Identifying patients who might benefit from scheduling and social support from community health workers or patient navigators is largely determined on a case-by-case basis and is resource intensive.

**Objective:**

This study aims to propose a novel algorithm to use scheduling data to identify complex scheduling patterns among patients with transportation and housing needs.

**Methods:**

We present a novel algorithm to calculate scheduling complexity from patient scheduling data. We define patient scheduling complexity as an aggregation of sequence, resolution, and facility components. Schedule sequence complexity is the degree to which appointments are scheduled and arrived to in a nonchronological order. Resolution complexity is the degree of no shows or canceled appointments. Location complexity reflects the proportion of appointment dates at 2 or more different locations. Schedule complexity captures deviations from chronological order, unresolved appointments, and coordination across multiple locations. We apply the scheduling complexity algorithm to scheduling data from 38 patients with breast cancer enrolled in a 6-month comorbidity management intervention at an urban hospital in the Washington, DC area that serves low-income patients. We compare the scheduling complexity metric with count-based metrics: arrived ratio, rescheduled ratio, canceled ratio, and no-show ratio. We defined an aggregate count-based adjustment metric as the harmonic mean of rescheduled ratio, canceled ratio, and no-show ratio. A low count-based adjustment metric would indicate that a patient has fewer disruptions or changes in their appointment scheduling.

**Results:**

The patients had a median of 88 unique appointments (IQR 60.3), 62 arrived appointments (IQR 47.8), 13 rescheduled appointments (IQR 13.5), 9 canceled appointments (IQR 10), and 1.5 missed appointments (IQR 5). There was no statistically significant difference in count-based adjustments and scheduling complexity bins (*χ*^2^_4_=6.296, *P*=.18). In total, 5 patients exhibited high scheduling complexity with low count-based adjustments. A total of 2 patients exhibited high count-based adjustments with low scheduling complexity. Out of the 15 patients that indicated transportation or housing insecurity issues in conversations with community health workers, 86.7% (13/15) patients were identified as medium or high scheduling complexity while 60% (9/15) were identified as medium or high count-based adjustments.

**Conclusions:**

Scheduling complexity identifies patients with complex but nonchronological scheduling behaviors who would be missed by traditional count-based metrics. This study shows a potential link between transportation and housing needs with schedule complexity. Scheduling complexity can complement count-based metrics when identifying patients who might need additional care coordination support especially as it relates to transportation and housing needs.

## Introduction

### Background

Patients with cancer frequently encounter complex treatment pathways, often characterized by challenges with coordinating and scheduling appointments at various specialty services and locations [1-3]. Previous studies have shown that the burden of scheduling and attending visits across multiple providers and specialties not only burdens patients, but also has ripple effects on families, work, and personal lives [4-6]. In a qualitative study with patients with metastatic breast cancer, patients pointed to the need for someone to coordinate appointments and a need for managing work-related barriers to attending appointments [7]. Furthermore, scheduling complexities do not fall on all patients equally. Patients facing social inequalities, such as unequal access to transportation, housing, and social support, face additional complexities in their cancer care appointments. For instance, patients with cancer without insurance, indicating financial vulnerability, are at high risk of no-show appointments [8,9]. A recent review illustrates that most research on multiappointment scheduling problems in oncology focus on solutions using metaheuristics and multiagent methods to ensure appointment adherence [2]. However, if scheduling complexities reflect underlying socioeconomic barriers, such solutions may not solve the structural issues.

To address structural access challenges around scheduling appointments, some health care institutions employ individuals such as community health workers (CHWs) or patient navigators, who play a pivotal role in guiding patients with cancer through their care journey by offering support for nonmedical needs [10]. CHWs and patient navigators have a wide variety of skills and can provide critical assistance coordinating appointment scheduling and overcoming barriers to attending care [4,11,12]. Patients who might benefit from this additional assistance are largely identified manually by CHWs or by care providers aware of possible challenges and social needs [13], or some clinics may assign all patients to CHWs to screen, a resource intensive process [14-16]. Workflows reliant on staff to identify those who might benefit the most from navigation can be time-consuming and resource-intensive, making it difficult to comprehensively identify patients in need of assistance. While ideally all patients would be offered navigation services, in light of staffing shortages and overall limited patient navigation resources, many institutions may be limited in who they can provide extra supportive services to [17]. A data-driven solution that alleviates burden from support staff (ie, reviewing charts to identify patient needs) or relying on clinician referrals would be ideal to effectively and efficiently allocate limited CHW and patient navigator resources.

### Potential of Scheduling Data

A potential way to identify patients with unmet transportation or housing needs is to use scheduling data to examine who is experiencing high scheduling complexities. Scheduling data for most cancer care is electronic, providing detailed data about when appointments are scheduled, cancelled, rescheduled, or no shows. This data is automatically recorded, and thus could be used to identify patients who are struggling to manage the complexity of cancer care. In past research, appointment data has primarily been used to optimize appointment scheduling for patient satisfaction and resource allocation [18-21]. Analyses tend to focus on developing and testing scheduling methods to best balance patient satisfaction (eg, wait times) with clinic resources. For example, using model simulations to optimize the scheduling of oncology visits and chemotherapy treatments [19], or optimizing scheduling rules based on chemotherapy infusion [21]. Other research using scheduling data examines the efficiency of appointment self-scheduling processes [22], optimizing scheduling for cost savings [20], and identifying ways to reduce wait times for patients [18]. A study designed an algorithm that used appointment data to identify patients’ primary care physician [23]. However, to our knowledge, researchers have yet to design tools for analyzing scheduling data to identify patients with possible unmet transportation or housing needs during their cancer care.

### Contributions

Our study used existing scheduling data to identify patients with complex scheduling patterns which may reflect unmet social needs in transportation and housing. We introduce a novel algorithm to calculate scheduling complexity from scheduling data using a sample of patients with breast cancer with initiating cancer treatment from a larger parent study intervening on comorbidity management [24]. Scheduling complexity is an aggregation of sequence, resolution, and facility components. Each component is motivated by the characteristics of scheduling data, an appointment’s anatomy, and possible outcomes. The scheduling complexity algorithm is then applied to the scheduling data of 38 patients with breast cancer as a case example. The resulting scheduling complexities are compared with count-based metrics and call notes between CHWs and patients to identify unmet transportation or housing needs.

## Methods

### Anatomy of an Appointment

**Figure 1.**
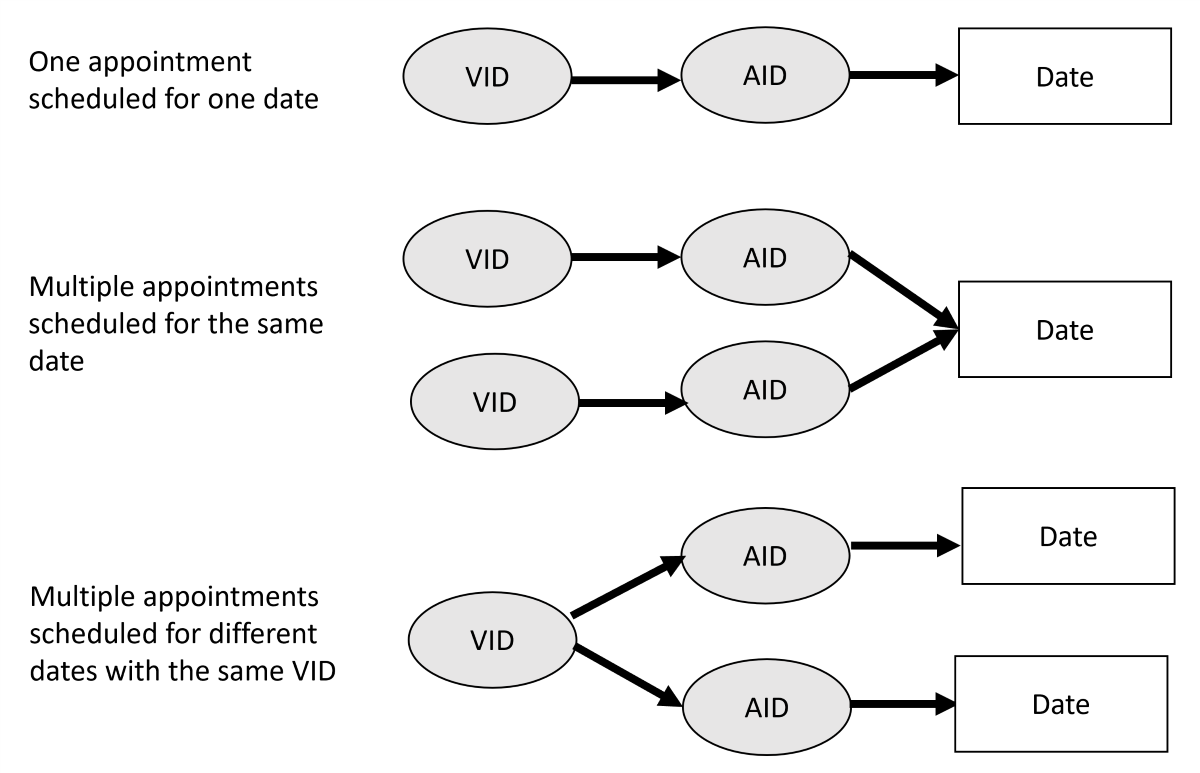
Illustration of appointment ID (AID) and visit ID (VID) possible scenarios.

Every appointment has a unique appointment identification (AID) and is scheduled on a specific date and time and scheduled for a specific date, time, and location. An appointment is scheduled for a specific visit reason and is associated with the corresponding visit identification (VID). Typically, 1 date will have 1 appointment scheduled with 1 associated VID, Figure 1 (top). Figure 1 is an illustration of AID and VID possible scenarios. Sometimes there can be multiple appointments with different VIDs scheduled for the same date. This is illustrated in an example patient schedule in Table 1. A magnetic resonance imaging (MRI) and mammogram are both scheduled for January 15, 2023. The MRI and mammogram appointments have different AIDs (AID-5 and AID-6 respectively) and VIDs (VID-4 and VID-5 respectively) because they have different reasons for visit and will be at different locations, 1 on the ground floor and 1 on the second floor of the hospital. There can also be multiple AIDs for different dates associated with the same visit reason and at the same location, VID, Figure 1 (bottom). A common example of this pattern is for daily treatments as illustrated in Table 1. There are 4 appointments for the same treatment at the same location with the same VID (VID-7) but with different AIDs (AID-9, AID-10, AID-11, and AID-12). All the AIDs are for the same treatment and at the same location and would have the same VID.

**Table 1.** Example individual patient scheduling temporal pattern.

VID^a^	AID^b^	Reason for visit	Location	Scheduled on	Scheduled for	Cancelled on	Rescheduled on	Arrived on
VID-1	AID-1	New consult	Hospital A - 2nd FL^c^	1/1/2023	1/5/2023	—^d^	—	1/5/2023
	AID-2	Colon screening	Hospital B - Ground	1/1/2023	2/1/2023	1/17/2023	—	—
VID-2	AID-3	Skin check	Hospital B - Ground	1/3/2023	1/10/2023	—	—	1/10/2023
VID-3	AID-4	Echocardiogram	Hospital A - Ground	1/5/2023	1/20/2023	—	—	1/20/2023
VID-4	AID-5	C50.912 MRI^e^	Hospital A - Ground	1/10/2023	1/15/2023	—	—	1/15/2023
VID-5	AID-6	LF^f^ breast mass - mammo	Hospital A - 2nd FL	1/15/2023	1/15/2023	—	—	1/15/2023
VID-6	AID-7	Follow up	Hospital A - 2nd FL	2/1/2023	2/20/2023	—	2/10/2023	—
VID-6	AID-8	Follow up	Hospital A - 2nd FL	2/10/2023	2/25/2023	—	—	2/25/2023
VID-7	AID-9	Treatment	Infusion center - 2nd FL	4/1/2023	4/5/2023	—	—	4/5/2023
VID-7	AID-10	Treatment	Infusion center - 2nd FL	4/1/2023	4/6/2023	—	—	4/6/2023
VID-7	AID-11	Treatment	Infusion center - 2nd FL	4/1/2023	4/7/2023	—	—	4/7/2023
VID-7	AID-12	Treatment	Infusion center - 2nd FL	4/1/2023	4/8/2023	—	—	4/8/2023

a VID: visit identification.

b AID:appointment identification.

c FL: floor.

d Not available.

e MRI: magnetic resonance imaging.

f LF: left.

### Appointment Action Outcomes

**Figure 2.**
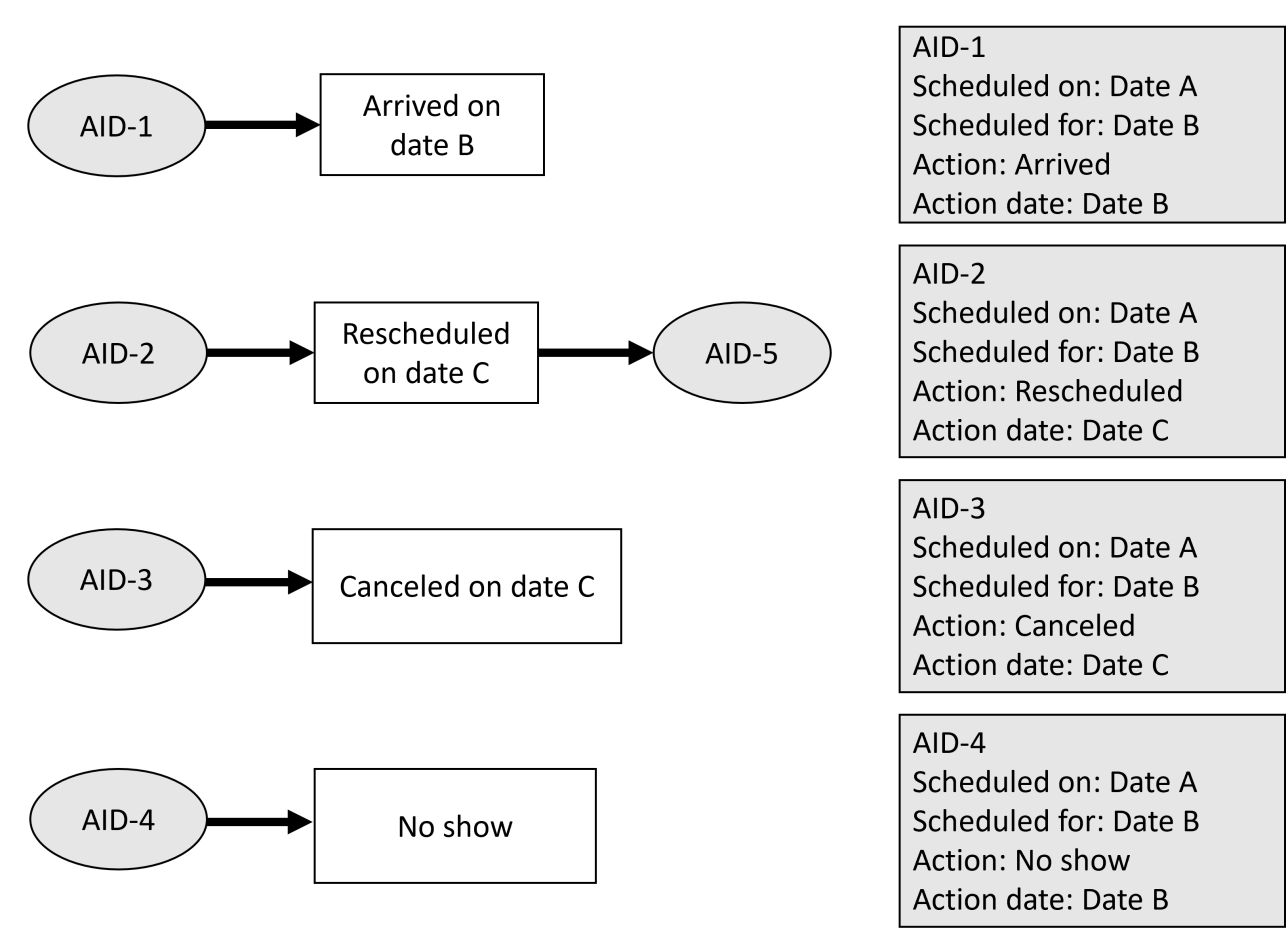
The 4 possible action outcomes for an appointment. AID: appointment ID.

There are 4 possible action outcomes for an appointment: arrived, rescheduled, canceled, or no show as illustrated in Figure 2. Figure 2 illustrates 4 possible action outcomes for an appointment. Arrived occurs when the patient arrives on the scheduled appointment date. Rescheduled occurs when the appointment needs to be rescheduled for another date. This can be due to multiple reasons, such as patient’s preference, medical necessity, financial or transportation issues, circumstances or system related factors, such as being bumped, unresolved insurance authorization, etc. Rescheduling an appointment will result in a new AID. An appointment can also be canceled by the patient or the hospital. For example, a provider could be unavailable due to illness or a scheduling conflict. Similarly, canceled appointments can be caused by a variety of patient or hospital system reasons. Finally, no show occurs when a patient doesn’t arrive to an appointment and does not cancel the appointment.

### Sources for Scheduling Complexities

#### Sequence Complexity: Appointment Ordering Sequence

We define schedule sequence complexity as the degree to which appointments are scheduled and arrived to in a nonchronological order. While there are several ways to define temporal complexities, we choose a queuing approach as it most closely aligns with patient scheduling experience [25,26]. As such schedules with low sequence complexity are those where appointments are scheduled and arrived in chronical order. This follows the general queuing rule of first in-first out: appointments scheduled first are arrived to first which minimizes the number of outstanding appointments at any given time. Schedules with more sequence complexity are those where appointments are scheduled and arrived to in a nonchronological order. Using the illustration in Table 1, AID-1 and AID-3 are examples of appointments scheduled and arrived in chronical order. AID-1 is scheduled before AID-3 and AID-1 is arrived to before AID-3. AID-4 and AID-5 are examples of appointments scheduled and arrived in nonchronological order. AID-4 is scheduled before AID-5 but the patient arrived to AID-5 before AID-4. This complexity can be caused by many factors such as appointments scheduled for the far future, canceling and rescheduling of appointments, or emergent appointments. These factors can increase schedule challenges both for the patient and scheduling systems.

#### Resolution Complexity: Unresolved Appointments

No shows or canceled appointments without rescheduling or reason can increase scheduling complexity in a patient’s care. Missing appointments leads to increase patient risk for cancer recurrence and mortality [27,28], and inefficiency for the health care system including lost revenue [29-31]. These unresolved appointments have no resolution, leaving uncertainty about potential delays in treatment and care. However, there are sometimes canceled appointments because of changes in treatment plans or no shows that are resolved through another action. Actions for these resolved appointments often co-occur with action dates for arrived or rescheduled appointments. Hence, we define resolution complexity as the number of no shows or canceled appointments on dates that do not co-occur with other action dates divided by the total number of no shows or canceled appointments.

#### Location Complexity: Appointments at Multiple Facilities

Having care at multiple facilities or locations can also increase scheduling complexities as this usually means more coordination and travel between facilities. Intuitively, a schedule with lower location complexity will have fewer facilities for care on the same day. A schedule with higher location complexity will more often require the patient to attend different facilities for care on the same day. Location complexity is calculated as the number of arrived dates involving 2 or more different locations divided by the total number of arrived dates.

### Calculating Scheduling Complexity

The algorithm for calculating a schedule’s scheduling complexity is described below (Textbox 1) . First, schedule data is separated into arrived and not arrived appointments, “ARRIVED” and “NONARRIVED” respectively. ARRIVED appointments are aggregated at the date level. For each AID in ARRIVED, if there exist other AIDs with scheduled on dates preceding the current AID’s scheduled on date and these subsequent appointments were attended after the current AID’s date, then the count of out-of-order occurrences is increased. Sequence complexity is calculated as the ratio of out-of-order counts to the total count of distinct arrived dates in ARRIVED. Next, for each AID in the NONARRIVED group, an action date is determined, representing the date when an appointment was either canceled, bumped, or scheduled but resulted in a no-show. If this action date does not appear in the dataset of ARRIVED appointments, then the count of unresolved cases is increased by 1. Resolution complexity is then computed as the ratio of unresolved counts to the total count of AIDs within the NONARRIVED group. Location complexity is calculated as the number of arrived dates in ARRIVED involving 2 or most different locations divided by the total number of arrived dates in ARRIVED. Finally, a composite metric scheduling complexity is the harmonic mean of sequence complexity, resolution complexity, and location complexity.

Textbox 1. **ALGORITHM: Deriving scheduling complexity**

**ARRIVED, NONARRIVED** ← Separate data into arrived and not arrived appointments

­

For each AID in **ARRIVED**:

 If there are other AID date that was made before current AID date and arrived to after current AID date

  Out of order count += 1

**sequence complexity** = out of order count / total count of unique arrived to dates in **ARRIVED**

­

For each AID in **NONARRIVED**:

 Action date = canceled or bumped date or scheduled date for no-show

 If Action date not in Arrived_data:

  unresolved += 1

**resolution complexity** = unresolved count / total count of nonArrived AIDs

­

For each arrived date with multiple AIDs in **ARRIVED**

 If AIDs are at different locations:

  Location count += 1

**location complexity** = location count / total number of arrived dates with multiple AIDs in **ARRIVED**

­

**scheduling complexity** = 3 / (1/sequence complexity + 1/resolution complexity + 1/facility complexity)

### Case Example and Study Background

To evaluate the use of scheduling complexity, we calculated the scheduling complexity for 38 patients with breast cancer who had hypertension or diabetes, as part of a larger health disparities project to support Black patients with cancer with comorbidities by mobile health and CHW support [24]. The 38 patients with breast cancer were enrolled in a 6-month comorbidity management intervention at an urban hospital in the Washington, DC area that serves low-income patients. This data was collected through the parent study whereby Black patients with breast and prostate cancer were recruited for a 6-month comorbidity management intervention. For tthis analysis, we focused on the association between scheduling patterns and social needs. Given significant differences in course of treatment by cancer site and time since diagnosis we limited the sample to those who had a diagnosis of breast cancer, all of whom had been diagnosed within the previous year. We further limited the sample to the 38 patients with breast cancer whom we had reliable appointment level data. The women in our sample were from an urban hospital in the Washington, DC area that primarily serves low-income patients. Black women with breast cancer in the DC area are a high priority sample, due to the increased mortality rate relative to White women [32].

### CHW Call Logs

In addition, as part of the parent study, we conducted a qualitative context analysis of CHWs’ call logs to identify social needs that arose throughout the study [33]. We used a deductive approach, first applying discrete categories from the health system screening tool focused on domains of food insecurity, housing instability, transportation, employment, financial strain, and utilities. Additional needs that were documented but did not fit into a predetermined category, such as access to wigs or special bras, were added as new codes using an inductive descriptive coding method to match social needs domain described by the Social Interventions Research and Evaluation Network (SIREN) [34].

### Data Analysis

For this pilot evaluation, we use 1 year of scheduling data for each patient starting from their date of diagnosis. Furthermore, 1 year of scheduling data was chosen because the majority of patients completed curative treatment within the first year after diagnosis. Sequence complexity, resolution complexity, location complexity, and scheduling complexity was calculated for each patient separately. In addition, we calculated count-based metrics: arrived ratio, rescheduled ratio, canceled ratio, and no-show ratio. We define an aggregated count-based adjustment metric as the harmonic mean of rescheduled ratio, canceled ratio, and no-show ratio. Count-based adjustments and scheduling complexities are stratified using quartiles and compared. We stratify patients into high, medium, and low complexities using the upper quartile, middle quartiles, or lower quartiles respectively. We used *χ^2^* test to compare our scheduling complexity metric to count-based adjustments because they are commonly used in first order analysis of scheduling data. All analysis was done in Python (version 3.0; Python Software Foundation).

### Ethical Considerations

This research was approved by the Georgetown University Institutional Review Board (STUDY00003543). This study was registered with ClinicalTrials.gov (NCT04836221). Written informed consent was obtained before conducting all study procedures, which allowed for secondary analysis of participant data without additional consent. A Health Insurance Portability and Accountability Act waiver and access to medical record data was included in the signed informed consent. All data included in this manuscript are deidentified and reported in aggregate. Data obtained through the study adhere to data protection and institutional review board standards as determined by the governing institution. Participants were compensated US $50 at the completion of the study.

## Results

### Schedule Descriptives

**Figure 3.**
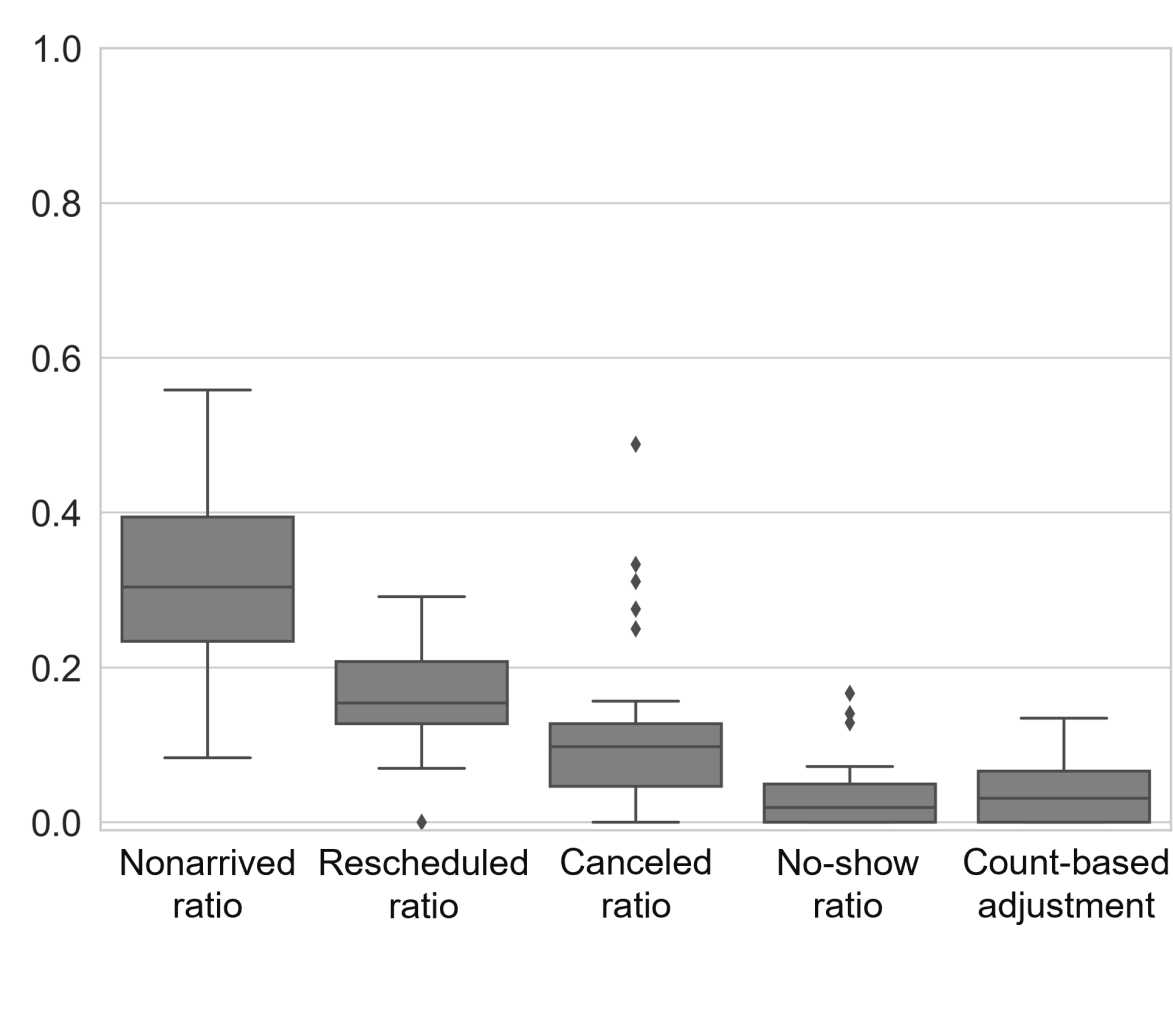
Summary boxplots of nonarrived ratio, rescheduled ratio, canceled ratio, and no-show ratio.

**Figure 4.**
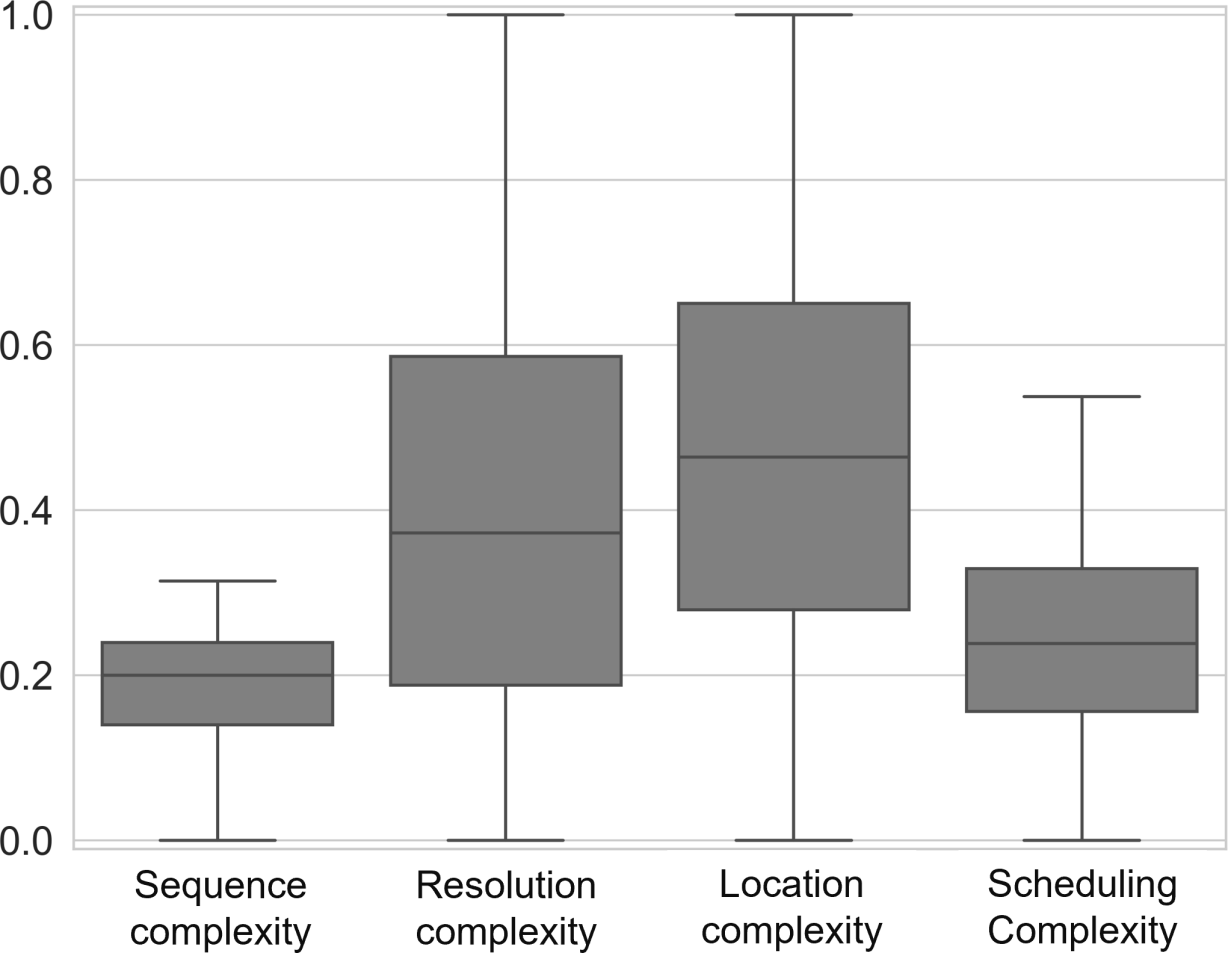
Summary boxplots of sequence complexity, resolution complexity, location complexity, and scheduling complexity.

A total of 38 female patients with breast cancer with an average age of 67.1 (SD 8.5), from 3 referring oncology providers had a median of 88 unique AID (IQR 60.3: first quartile [Q1]=59, third quartile [Q3]=119.3), 62 arrived appointments (IQR 47.8: Q1=38.7, Q3=86.5), 13 rescheduled appointments (IQR 13.5: Q1=7.5, Q3=21), 9 canceled appointments (IQR 10.0: Q1=3, Q3=13), and 1.5 missed appointments (IQR 5: Q1=0, Q3=5). The median nonarrived ratio was 0.304 (IQR 0.161: Q1=0.233, Q3=0.394). The median rescheduled ratio was 0.154 (IQR 0.080: Q1=0.127, Q3=0.207). The median canceled ratio was 0.098 (IQR 0.081: Q1=0.046, Q3=0.127) and the median no-show ratio was 0.019 (IQR 0.049: Q1=0, Q3=0.049). Figure 3 is a summary boxplot of nonarrived, rescheduled, canceled, and no-show ratios with nonarrived ratio being the largest. The median sequence complexity was 0.200 (IQR 0.100: Q1=0.140, Q3=0.240). Figure 4 is a summary boxplot of sequence, resolution, location, and scheduling complexities with location complexity being the largest. The median resolution complexity was 0.372 (IQR 0.398: Q1=0.188, Q3=0.586). The median location complexity was 0.464 (IQR 0.371: Q1=0.279, Q3=0.650). Finally, the median scheduling complexity was 0.239 (IQR 0.173: Q1=0.156, Q3=0.329).

### Comparison of Count-Based Adjustments With Scheduling Complexity

A total of 18 patients exhibited medium count-based complexities, 10 patients exhibited high count-based complexities, and 10 patients exhibited low count-based complexities. Similarity, 18 patients exhibited medium scheduling complexities, 10 patients exhibited high scheduling complexities, and 10 patients exhibited low scheduling complexities. There was no statistically significant difference in count-based adjustments and scheduling complexity bins (χ^2^_4_=6.296, *P*=.18). Table 2 shows the count-based and scheduling complexities were the same for 16 patients, 11 of which had both medium scheduling and count-based complexities. Furthermore, 5 patients exhibited high scheduling complexity with low count-based adjustments and 2 patients who exhibited high count-based adjustments with low scheduling complexity.

**Table 2.** Correlation of scheduling and count-based complexities binned by low, medium, and high.

Scheduling complexity		Count-based adjustments		
		Low	Medium	High
Low		3	5	2
Medium		2	11	5
High		5	2	3

**Figure 5.**
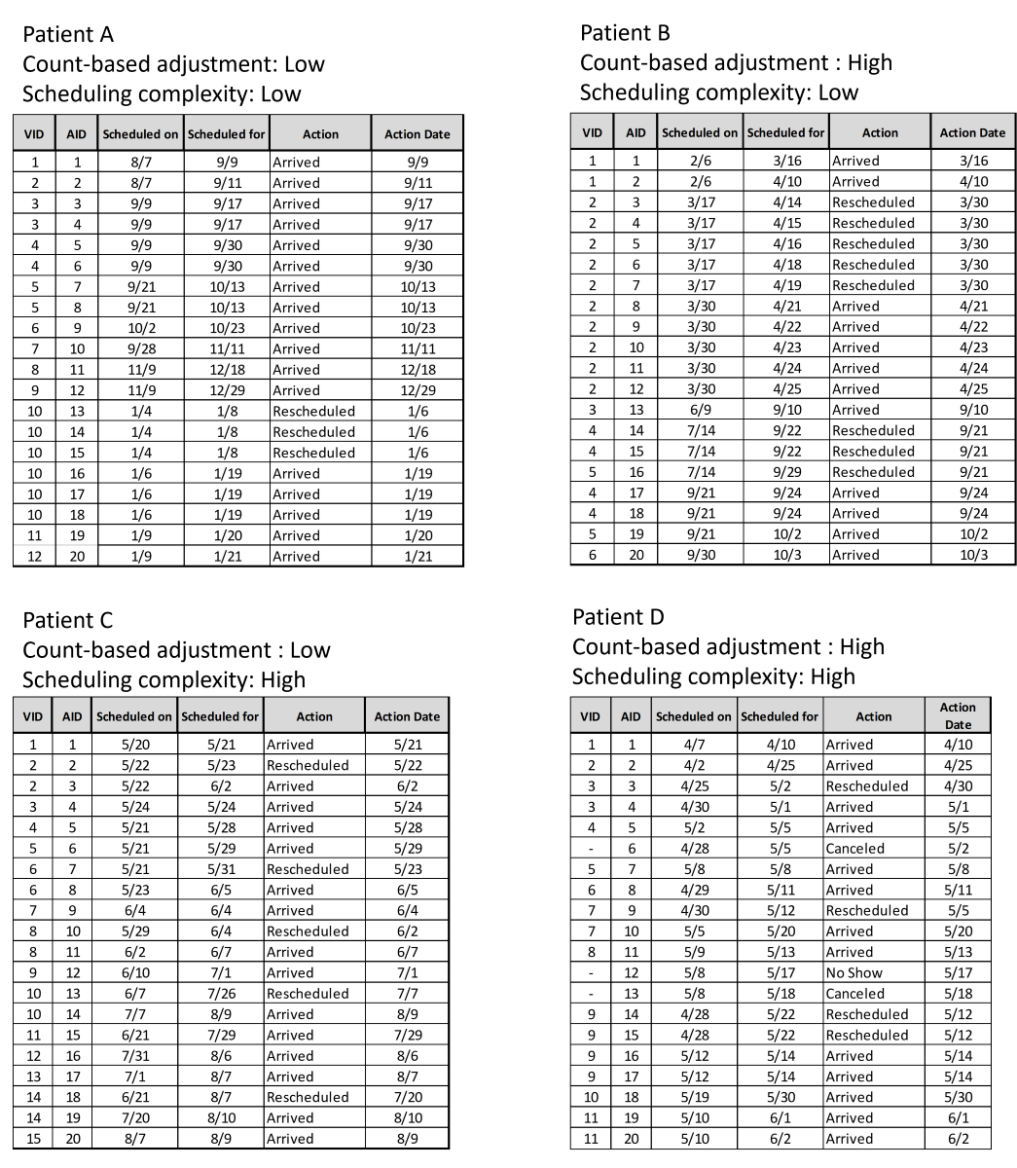
Examples of high scheduling complexity and low count-based adjustments, high count-based adjustments and low scheduling complexity, low scheduling complexity and low count-based adjustments, and high scheduling complexity and high count-based adjustments. AID: appointment identification; VID: visit identification.

In addition, Figure 5 gives examples of high scheduling complexity and low count-based adjustments, high count-based adjustments and low scheduling complexity, low scheduling complexity and low count-based adjustments, and high scheduling complexity and high count-based adjustments. Patient A had both low count-based adjustments and low scheduling complexity. Patient A’s schedule is a good example of appointments following the first-in first-out pattern, that is appointments scheduled first will be arrived to first. In addition, Patient A only had 3 rescheduled appointments. Patient B had high count-based adjustments but low scheduling complexity; 40% (8/20) of Patient B’s AID were rescheduling actions which likely contributing to a high count-based adjustments. However, Patient B had low scheduling complexity because these rescheduling actions occurred only on 2 separate days and followed a first-in first-out sequence. Patient C had low count-based adjustments and high scheduling complexity. Although Patient C had few rescheduling actions (resulting in a low count-based adjustments), her appointments were largely scheduled and arrived to not in chronical order (resulting in a high scheduling complexity). Patient D had both high count-based adjustments and high scheduling complexity. In addition to nonchronical ordering of action, Patient D had 2 canceled and 1 no-show appointment action outcomes.

### Context From Call Logs: Transportation and Housing Needs

A total of 15 patients specifically indicated transportation or housing insecurity issues. Transportation concerns included “legally blind and worried about metro access,” “[patient] feeling unsafe on metro,” “transportation challenges to and from appointments,” “extensive travel requirements,” “making medical transportation rides,” and “needing transportation assistance.” Housing concerns included “help with finding affordable housing options,” concerns related to advocating for tenant rights, home repair needs, “[patient] moving in with relative for a few months to save money,” “having to find temporary housing while home is being repaired,” “help finding rental assistance programs.” Additional examples of identified social needs included a demanding work schedule, complexities with an eye surgery, and the additional responsibility of caring for an ill mother. Scheduling complexity was more sensitive to housing and transportation needs. 86.7% (13/15) of patients specifically indicated transportation or housing insecurity issues were identified as medium or high scheduling complexity compared with 65.2% (15/23) of patients who did not specifically indicate transportation or housing insecurity issues. On the other hand, 60% (9/15) of patients specifically indicated transportation or housing insecurity issues were identified as high with count-based adjustments compared with 82.6% (19/23) of patients who did not specifically indicate transportation or housing insecurity issues Table 3.

**Table 3.** Percentage of patients with medium or high complexities by transportation or housing insecurity needs.

Complexity type	Indicated transportation or housing needs (n=15), n (%)	Did not indicate transportation or housing needs (n=23), n (%)
Count-based adjustments	9 (60)	19 (82.6)
Scheduling complexity	13 (86.7)	15 (65.2)

## Discussion

### Principal Findings

Scheduling complexity stratification provides a novel lens to complement traditional count-based metrics for analyzing scheduling data. The results show that scheduling complexity can identify patients with complex but nonchronical scheduling behaviors missed by traditional count-based metrics. In addition, the study highlights that resolution and location complexity can also serve as an indicator for additional care requirements.

### Comparison With Previous Work

This study shows a potential link between transportation and housing needs with schedule complexity. This study reinforces previous research relating social risk factors and schedule complexities [4,9]. Our results complement these findings as it relates to transportation and housing needs and highlights the potential use of the scheduling complexity algorithm to identify patients who might benefit from additional CHW support. Through earlier identification from CHWs, scheduling complexity could help narrow inequities in cancer-care which emerge from social needs. Future studies are needed to better understand temporal sensitivity of this approach, or how quickly in-need patients could be identified.

### Support for CHW and Patient Navigators

By examining the temporal patterns of health care use, we gain a more comprehensive view of patients’ experiences. Instead of relying solely on infrequent screeners, scheduling complexity can give CHWs and patient navigators a more “real-time” view of patients who might require more support in managing their health care journey, for example patients with changing, complex, and distributed care and changes in living conditions or social needs. In addition, scheduling complexity could also be used to identify care plans that might involve more complexity and preemptively identify patients that might need more support. This data-driven approach can help complement the often manual process for identifying patients who might benefit from additional assistance, potentially affording CHWs and patient navigators more time to directly care and help patients [13]. Additional research is needed to evaluate the utility of this algorithm in near “real-time” applications for CHWs.

### Limitations and Future Directions

There are several limitations to consider in this pilot work. First, this research is constrained by its retrospective analysis design, relying on historical data and records. Second, a naïve weighting approach was taken in which sequence, resolution, and location complexity are weighted equally in the algorithm. These components might require different weights depending on circumstances. For example, for cancer care at integrated cancer centers, location complexity would probably be less important than sequence and resolution complexities. Third, the study exclusively focuses on the scheduling system of a single urban cancer institute. As such, the results only reflect the central tendencies given the specific conditions and scheduling pattern characteristics of our patient cohort. The generalizability this approach and interpretation of the metrics to other health care systems and patient conditions will need to be explored. Fourth, while this approach has provided insights, it does not fully capture the entirety of factors that contribute to scheduling intricacies, such as resource allocation, patient preferences, and staff availability. Further validation is needed beyond this case example, particularly with a larger patient sample to better capture variations in the scheduling complexity data. Nevertheless, this method could complement other approaches such as patient and scheduler interviews. This work should also be explored in a larger sample size to further explore our hypotheses generated about scheduling complexity.

### Conclusion

Patients facing complex health care journeys often experience significant impacts on various aspects of their lives, including family dynamics and work commitments. While there was no statistically significant difference in count-based adjustments and scheduling complexity bins, we showed that scheduling complexity can uniquely identify patients with complex and nonchronical schedule behaviors. We highlight the potential use of scheduling complexity in identifying patients who might need care coordination support especially as it relates to transportation and housing needs.

## Abbreviations

AID: appointment identification
CHW: community health worker
MRI: magnetic resonance imaging
Q1: first quartile
Q3: third quartile
VID: visit identification
